# Diffuse Leukoencephalopathy and Subacute Parkinsonism as an Early Manifestation of Systemic Lupus Erythematosus

**DOI:** 10.1155/2013/367185

**Published:** 2013-11-28

**Authors:** Gary G. Tse, Alberto S. Santos-Ocampo, Dominic C. Chow, Aaron M. McMurtray, Beau K. Nakamoto

**Affiliations:** ^1^Department of Medicine, University of Hawaii, Honolulu, HI 96813, USA; ^2^Straub Clinics and Hospital, Honolulu, HI 96813, USA; ^3^Department of Neurology, Harbor-UCLA Medical Center, Torrance, CA 90502, USA

## Abstract

Parkinsonism in SLE is rare. Diffuse leukoencephalopathy is equally uncommon and is associated with a poor prognosis. We present a single case of a 50-year-old Filipino man who presented with a generalized discoid rash after starting lisinopril. The rash persisted despite discontinuation of lisinopril, and over the next three months, he developed rapidly progressive parkinsonism. Brain MRI showed symmetric confluent T2-hyperintensities involving the white matter and basal ganglia. Four of the 11 American College of Rheumatology criteria for the classification of SLE were met. A rheumatologist made a diagnosis of SLE with cutaneous and central nervous system involvement. Significant neurologic and radiologic improvement occurred following treatment with IV steroids followed by a prolonged taper. This report highlights a case of subacute parkinsonism with a diffuse leukoencephalopathy as an early manifestation of SLE which resulted in a good recovery following treatment with only immunosuppressive therapy.

## 1. Introduction

Central nervous system (CNS) involvement is present in up to 70% of individuals with systemic lupus erythematosus (SLE) and can present with an acute confusional state, demyelinating syndrome, stroke, seizures, or cognitive dysfunction [1]. Radiologic abnormalities associated with CNS lupus include demyelinating plaques, myelitis, ischemic or hemorrhagic stroke, dural venous sinus thrombosis, rhombencephalitis, and cerebral atrophy [2]. We report a single case of subacute parkinsonism with a diffuse leukoencephalopathy on brain MRI as an early manifestation of SLE.

## 2. Report of a Case

**Figure 1:**
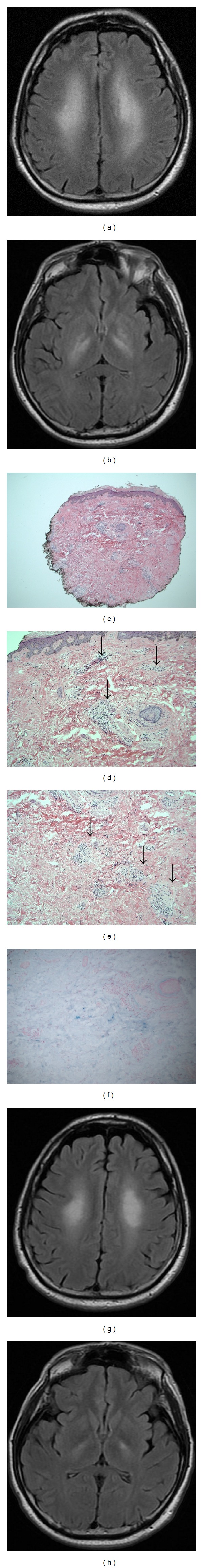
50-year-old male presenting with diffuse, confluent leukoencephalopathy and subacute parkinsonism. Axial brain MRI FLAIR sequence on initial presentation showing extensive confluent symmetric hyperintensities ((a) and (b)). Gross skin punch biopsy from right knee (c) shows superficial ((d), arrows) and deep perivascular dermatitis ((e), arrows) with focally increased dermal mucin highlighted by Alcian blue stain (f). Repeat axial brain MRI FLAIR sequence after 5 months demonstrating radiographic improvement following immunosuppressive therapy ((g) and (h)).

A 50-year-old Filipino male with a history of hypertension developed a discoid, photosensitive, and hyperpigmented rash on sun-exposed areas of his face, arms, and legs in March 2012 shortly after starting lisinopril. Lisinopril was discontinued in April 2012 without resolution of the rash, prompting consultation with a dermatologist who suspected discoid lupus and ordered screening lupus labs. Complete blood count (CBC) was normal. Erythrocyte sedimentation rate (ESR) was 76 mm/hour, anti-nuclear antibody (ANA) 1 : 320 in a homogenous and fine speckled pattern, anti-histone and anti-RNP antibodies were positive, and complement C50, C3, and C4 were low. Anti-double-stranded DNA (anti-ds DNA) and anti-smith (anti-Sm) antibodies were negative. Initial impression was drug-induced lupus. In July 2012, he was admitted for recurrent falls. Neurological examination was notable for reduced degree of facial expression (hypomimia), weak and soft speech (hypophonia), and a mild shuffling gait. Muscle strength was normal (i.e., graded 5 in all muscle groups by Medical Research Council scale for motor strength). He denied feeling depressed. Cerebrospinal fluid (CSF) analysis revealed 0 WBC/uL, 3 RBC/uL, protein was 88 mg/dL, and glucose was 36 mg/dL. Contrast-enhanced brain MRI revealed symmetric confluent periventricular white matter hyperintensities on T2-weighted sequences associated with restricted diffusion on diffusion-weighted sequences and corresponding hypointense signal on Apparent Diffusion Coefficient map markedly more severe than the mild small-vessel ischemic changes which would be seen in a similarly aged individual with a history of hypertension (Figures 1(a) and 1(b)). There was mild symmetric dural enhancement that was attributed to the lumbar puncture. Head MRA was normal. He was discharged but was readmitted two weeks later reporting progressive weakness. General physical examination revealed a discoid, hyperpigmented rash on sun-exposed areas of his face, arm, and legs. No oral ulcers were noted. His peripheral joints were normal. Lungs were clear to auscultation, and no friction rub was present. Neurological examination revealed an awake and alert individual who was oriented to person, place, and time with slowed mentation (bradyphrenia). He had marked hypomimia, hypophonia, symmetric bradykinesia, and cogwheel rigidity. No tremor was present. He was unable to arise from bed without maximal assistance and was unable to initiate a step despite normal motor strength. Part III, Unified Parkinson's Disease Rating Scale (UPDRS) was 47 (UPDRS range is 0 to 56, where 0 is normal and 56 is the most severe motor disability related to parkinsonism). CBC during this admission was notable for a white blood cell count that dropped to 2.7 × 10^9^/L × 10^9^/L, absolute lymphopenia of 0.59 × 10^9^/L (normal range 0.7–4.5 × 10^9^/L), and thrombocytopenia of 111 × 10^9^/L (normal range 150–450 × 10^9^/L). Repeat ESR was 91 mm/hr, ANA 1 : 640 in a homogenous pattern, anti-RNP and anti-histone antibodies positive, and complement C3 low. Repeat anti-ds and anti-sm antibodies were negative. Evaluation for toxic encephalopathies (urine toxicology, alcohol, toluene, arsenic, mercury, and lead levels), metabolic disorders (vitamin B12, folate, copper, ceruloplasmin, vitamin E, and TSH), autoimmune disorders (anti-SSA, anti-SSB, angiotensin converting enzyme, anti-neutrophil cytoplasmic antibodies (cytoplasmic and perinuclear), rheumatoid factor, phospholipid antibodies, beta-2 glycoprotein antibodies, lupus anticoagulant, immunofixation electrophoresis, cryoglobulin, TSH receptor, thyroid peroxidase, thyroglobulin antibodies, paraneoplastic antibodies, and NMDA antibodies), infections (human immunodeficiency virus, syphilis, lyme, human T-lymphotropic virus, JC virus, and hepatitis C virus), and inherited mitochondrial and metabolic disorders (CK, lactic acid, very long chain fatty acids, plasma and urine amino acids, urine organic acids, homocysteine, total cholesterol, total hexosaminidase, hexosaminidase A, and evaluation for lysosomal disorders) was negative. Repeat CSF analysis revealed 3 WBC/uL, 98 RBC/uL, protein being 61 mg/dL, and glucose being 41 mg/dL. Repeat contrast-enhanced brain MRI was unchanged from prior MRI. Chest, abdomen, pelvic CT, and scrotal ultrasound was normal. EEG revealed diffuse symmetric slowing without periodic triphasic waves. Repeat CBC during this admission revealed persistent leukopenia with a white blood cell count of 2.8 × 10^9^/L  (normal range 3.8–11.2 × 10^9^/L). The patient met 4 of 11 criteria based on the 1997 American College of Rheumatology revised criteria for classification of SLE by the presence of an abnormal ANA titer, discoid rash, photosensitivity, and a hematologic disorder (leukopenia on at least two occasions) [3]. Biopsy of a discoid skin lesion revealed superficial and deep perivascular dermatitis with focally increased dermal mucin highlighted by Alcian blue stain which suggested SLE versus Mixed Connective Tissue Disease (Figures 1(c)–1(f)). Patient was referred to rheumatology and was diagnosed with SLE with cutaneous and central nervous system involvement. He was started on intravenous methylprednisolone 120 mg (2 mg/kg/dose) daily for 3 days, then 80 mg daily for 3 days, and then 60 mg daily for 3 days. He was converted to oral prednisone and gradually tapered over the next 4 months to 10 mg of prednisone daily. No dopaminergic agents were used. Four months after his hospital discharge, his rash resolved and neurologic examination improved significantly. He had mild hypomimia, hypophonia, bradykinesia, and bilateral resting leg tremor with a normal gait. UPDRS (Part III) was 6. The leukopenia, thrombocytopenia, ESR, and hypocomplementemia normalized, although anti-histone antibodies were persistently elevated. There was marked radiologic improvement with resolution of the diffusion restriction (Figures 1(g) and 1(h)). He continues to be alert, oriented to person, place, and time, ambulatory, and functionally independent with unchanged neurologic and UPDRS (Part III) examination 9 months after his hospital discharge on 10 mg of prednisone daily.

## 3. Discussion

To our knowledge, this is the first reported case of diffuse leukoencephalopathy associated with a clinical presentation of subacute parkinsonism as an early manifestation of SLE. Parkinsonism is an uncommon neurologic manifestation of SLE, and movement disorders are not included in the 1997 American College of Rheumatology revised criteria for classification [3]. A few cases of parkinsonism have been reported in the setting of SLE and can occur in childhood or adults [4–8]. Most cases occurred in individuals with an established diagnosis of SLE. Parkinsonism is uncommon as the initial presentation of SLE [5, 6]. While the presence of systemic symptoms and signs, altered mental status, or subacute progressive parkinsonism assists with the diagnosis, these features are not invariably present, and cases of parkinsonism preceding the diagnosis of SLE by up to 10 years have been reported [7]. Brain MRI can assist with the diagnosis of SLE-associated parkinsonism, but the findings can either be normal or include nonspecific hyperintensities involving the deep gray matter and corona radiata on T2-weighted sequences [9, 10].

Only two cases of diffuse symmetric leukoencephalopathy have been described in SLE [11, 12]. Both cases presented with headaches associated with a rapidly progressive encephalopathy and raised intracranial pressure on lumbar puncture which was unresponsive to immunosuppressive therapy and ultimately ended in death. In our patient, the unusual presentation of a leukoencephalopathy on neuroimaging and subacute onset of parkinsonism as an early manifestation of new onset SLE led to an extensive evaluation and delayed diagnosis and treatment.

While the mechanism of SLE-associated parkinsonism is unknown, there is evidence that anti-dopaminergic antibodies may be involved [13]. Kunas et al. reported the presence of anti-dopaminergic antibodies in the serum of an individual with a prior diagnosis of SLE who presented with rapidly progressive parkinsonism [13]. These antibodies were repeatedly demonstrated during three years of followup and were not detected in 10 SLE controls (5 with and 5 without CNS involvement). We hypothesize that the symmetric confluent leukoencephalopathy associated with SLE in our patient may be due to immune-mediated cytotoxic edema [2, 10]. Most cases of SLE-associated parkinsonism improve following initiation of immunosuppressive therapy consistent with this hypothesis [5, 8]. While immunosuppressive therapy is usually the cornerstone of therapy for SLE-associated parkinsonism, supplementation with carbidopa/levodopa can be helpful, but symptomatic improvement is less robust [5, 8]. Radiographic findings, however, seem to lag behind changes on clinical examination.

In conclusion, our case highlights that SLE should be considered in the radiologic differential diagnosis of a diffuse symmetric leukoencephalopathy. This radiologic finding is not necessarily associated with a poor prognosis. Recognition of leukoencephalopathy associated with a clinical presentation of subacute, rapidly progressive parkinsonism as a possible early presentation of SLE will facilitate diagnosis and treatment.
